# UV/Vis Light Induced Degradation of Oxytetracycline Hydrochloride Mediated by Co-TiO_2_ Nanoparticles

**DOI:** 10.3390/molecules25020249

**Published:** 2020-01-07

**Authors:** Soukaina Akel, Redouan Boughaled, Ralf Dillert, Mohamed El Azzouzi, Detlef W. Bahnemann

**Affiliations:** 1 Institut für Technische Chemie, Leibniz Universität Hannover, Callinstr. 3, D-30167 Hannover, Germany; r.boughaled@gmail.com (R.B.); dillert@iftc.uni-hannover.de (R.D.); 2 Laboratory of Spectroscopy, Molecular Modeling, Materials, Nanomaterials, Water and Environment, (LS3MN2E) Faculty of Sciences, University Mohammed V. BP 1014, Rabat 10000, Morocco; elazzouzim@gmail.com; 3 Laboratorium für Nano-und Quantenengineering, Leibniz Universität Hannover, Schneiderberg 39, D-30167 Hannover, Germany; 4 Laboratory “Photoactive Nanocomposite Materials”, Saint-Petersburg State University, Ulyanovskaya Street 1, Peterhof, Saint-Petersburg 198504, Russia

**Keywords:** photocatalysis, Co-TiO_2_ catalyst, oxytetracycline hydrochloride, solvothermal synthesis, water treatment

## Abstract

Pharmaceuticals, especially antibiotics, constitute an important group of aquatic contaminants given their environmental impact. Specifically, tetracycline antibiotics (TCs) are produced in great amounts for the treatment of bacterial infections in both human and veterinary medicine. Several studies have shown that, among all antibiotics, oxytetracycline hydrochloride (OTC HCl) is one of the most frequently detected TCs in soil and surface water. The results of the photocatalytic degradation of OTC HCL in aqueous suspensions (30 mg·L^−1^) of 0.5 wt.% cobalt-doped TiO_2_ catalysts are reported in this study. The heterogeneous Co-TiO_2_ photocatalysts were synthesized by two different solvothermal methods. Evonik Degussa Aevoxide P25 and self-prepared TiO_2_ modified by the same methods were used for comparison. The synthesized photocatalysts were characterized by X-ray powder diffraction (XRD), Raman spectroscopy, transmission electron microscopy (TEM), UV/vis diffuse reflectance spectroscopy (DRS), and N_2_ adsorption (BET) for specific surface area determination. The XRD and Raman results suggest that Ti^4+^ was substituted by Co^2+^ in the TiO_2_ crystal structure. Uv/visible spectroscopy of Co-TiO_2_-R showed a substantial redshift in comparison with bare TiO_2_-R. The photocatalytic performance of the prepared photocatalysts in OTC HCL degradation was investigated employing Uv/vis spectroscopy and high-performance liquid chromatography (HPLC). The observed initial reaction rate over Co-TiO_2_-R was higher compared with that of Co-TiO_2_-HT, self-prepared TiO_2_, and the commercial P25. The enhanced photocatalytic activity was attributed to the high surface area (153 m^2^·g^−1^) along with the impurity levels within the band gap (2.93 eV), promoting the charge separation and improving the charge transfer ability. From these experimental results, it can be concluded that Co-doping under reflux demonstrates better photocatalytic performances than with the hydrothermal treatment.

## 1. Introduction

The growing demand for clean water sources has become an important issue worldwide owing to increasing water pollution by, for example, pharmaceuticals, personal care products, and endocrine disruptors. Among a wide variety of pharmaceutical compounds, antibiotics, owing to their extensive use in human and veterinary medicine, may cause environmental impacts, disturbing the function of the ecosystem by developing antibiotic-resistant pathogens that are of potential risk for human health after incorporation with drinking water, and vegetables or fruits irrigated with contaminated water [1,2,3].

Tetracyclines (TCs), as the second most commonly known antibiotic in production and use, have been used as bacteriostatic agents for treating infections in both humans and animals, and as fungicides in fruit tree [4,5]. Oxytetracycline hydrochloride (OTC HCl), a common member of tetracyclines (TCs), is a broad-spectrum antibiotic frequently employed in veterinary medicine and agriculture. Although the concentrations of the antibiotics released from the environmental matrix to aquatic systems are extremely low (μg·L^−1^ to mg·L^−1^), OTC HCl recognized as an emerging pollutant may cause serious risks to human health and ecosystems [6].

Owing to its chemical stability and antibiotic property, residues of OTC HCl released in the aquatic environment cannot be removed by conventional water treatment processes [7]. Therefore, advanced oxidation processes (AOPs) have been considered as one of the most effective technologies to clean off the aquatic environment from this contaminant of emerging concern [2,8]. So far, the photodegradation of tetracycline hydrochloride (TC HCl) has been reported using the photo-Fenton process [4,9] and ozonation [10,11]. Degradation of OTC has also been studied by the action of ultra-violet (UV) light, UV/H_2_O_2_, and UV activation of persulfate (UV/PS) [6,12,13]. However, there are numerous limitations such as the formation of byproducts, selective functional, photosensitive groups, and contact time [3]. Therefore, treatment processes using heterogeneous photocatalysis with TiO_2_ have received enormous attention in the recent years and have been employed in water treatment as a promising method for removal and mineralization of organic contaminants such as tetracyclines that are present in the aquatic environment [5,14,15,16,17,18].

Photocatalysis using semiconductors has gained an important place among the AOPs. Notably, TiO_2_ has received much more attention thanks to the high oxidizing power of its holes, as well as its photostability, non-toxicity, and low cost [19]. However, TiO_2_, owing to its intrinsic wide band gap (3.2 eV for anatase, 3.0 eV for rutile), can be only activated under UV light. Moreover, 90% of the photogenerated electrons and holes recombine in 10 ns [20]. These drawbacks are still a major limitation for the widespread application of TiO_2_.

In this respect, investigations of the visible light responsivity of TiO_2_ have been developed via TiO_2_ doping by low amounts of cations [21]; anions [22,23]; and transition metals such as Fe, Cu, Mn, Ni, Cr, Zn, and Co [21,22,23,24,25,26,27]. Among various transition metals (i.e., Pt, Ag, Au, Ru, and Pd) [28,29,30,31] and transition metal oxides (i.e., Cu_2_O, α-Fe_2_O_3_) [32,33,34], cobalt [35,36] and cobalt oxides [37,38,39,40] as an interesting low-cost and earth-abundant mineral have attracted tremendous attention for the development of visible light active TiO_2_ materials. Cobalt-doped TiO_2_ photocatalysts have been synthesized by various methods, such as the sol/gel technique [36,41,42,43,44,45,46,47,48,49,50], hydrothermal treatment [51,52,53,54], impregnation method [55,56], precipitation process [57] for the photocatalytic degradation of methylene blue [57,58], rhodamine B [58], methylene orange [57], phenol [41], 2-chlorophenol [41], and so on.

Among these preparation methods, hydrothermal synthesis as a well-known preparation method is environmentally friendly because the reaction proceeds in a closed system; the composition of the products is well controlled; and the materials prepared employing this method are well crystallized and have smaller particle size, positively affecting the thermal stability and the photocatalytic activity. For example, Jiang, et al. [59] synthesized cobalt-doped TiO_2_ by a one-step hydrothermal method for the photodegradation of phenol under visible light. They found that the catalyst doped with 0.3 wt.% shows the highest photocatalytic activity, and they contributed that to the high visible light response by inducing impurity states within its band gap. Rashad, et al. [60] reported the photocatalytic degradation of methylene blue under UV light in the presence of cobalt-doped TiO_2_ prepared using a hydrothermal treatment with a post-annealing temperature process at 500 °C. They observed a decreased in the surface area, a blue shift of the band gap with a slight enhancement in the photocatalytic activity by adding Co ions. Controversially, Castro, et al. [51] synthesized Co-doped TiO_2_ powders using hydrothermal synthesis. A mixed oxidation state of cobalt ions, that is, Co^3+^ and Co^4+^, was deduced. Even if a decrease in the band gap was observed, no photocatalytic degradation of diquat under UV light was detected. They concluded that both the doped-metal content and valence of the doping ions are crucial factors that strongly determine the photocatalytic activity of the materials.

Reflux synthesis as an alternative low-temperature process is much more beneficial because of the lower equipment cost and simplicity. However, the doping of TiO_2_ with cobalt using this method had just a few reports [61].

Although Co-doped TiO_2_ nanoparticles (NPs) have been extensively studied previously, there are conflicting results on the effects of cobalt ion doping on the photoactivity of TiO_2_. For example, Choi, et al. reported that doping with Co^3+^, among various transition metals using the sol/gel method, decreased the photoactivity for the degradation of CHCl_3_ under UV irradiation [21]. In 2010, the same group conducted a study on 13 different metal ions-doped TiO_2_ with a sol/gel procedure and indicated that Co-TiO_2_ material had a slight increase in the observed rate constant of methylene blue degradation. They also concluded that it is difficult to correlate between the physicochemical properties such as light absorption and the visible light photocatalytic activities of the studied metal-doped TiO_2_ materials [43]. The investigation of Bouras, et al. indicated that Co-doping applying a sol/gel synthesis had a detrimental effect on the photocatalytic behavior of TiO_2_ for the photocatalytic degradation of basic blue [44]. In contrast, other scientific groups reported that cobalt-modified TiO_2_ synthesized by wet impregnation methods seems to increase the photocatalytic activity [55,56]. Additionally, earlier studies have been mostly done on Co-doped TiO_2_ thin films, and the main focus has commonly been made on the ferromagnetic properties of these catalysts [45,53,62,63,64].

The different synthesis methods employed to synthesize cobalt-doped TiO_2_ along with the different types of substrates utilized for photocatalytic degradation examinations create a varying set of data that can often become controversial. These diverging results create doubts about the factual influence of the cobalt doping on the photocatalytic activity. Therefore, Co-doped TiO_2_ materials have been synthesized by means of two different methods, namely reflux and hydrothermal, to investigate the effect of incorporating cobalt cations in the TiO_2_ matrix, as well as their photocatalytic activity under UV/visible irradiation. Although Co-doped TiO_2_ NPs can be synthesized easily employing the reflux method, hardly any reports discussing its photocatalytic activity have been published. Until now, and to the best of the authors’ knowledge, the UV/vis light-induced photocatalytic degradation of oxytetracycline hydrochloride (OTC HCl) in the presence of Co-doped TiO_2_ NPs has not been studied.

## 2. Results

### 2.1. Photocatalysts Characterizations

**Figure 1:**
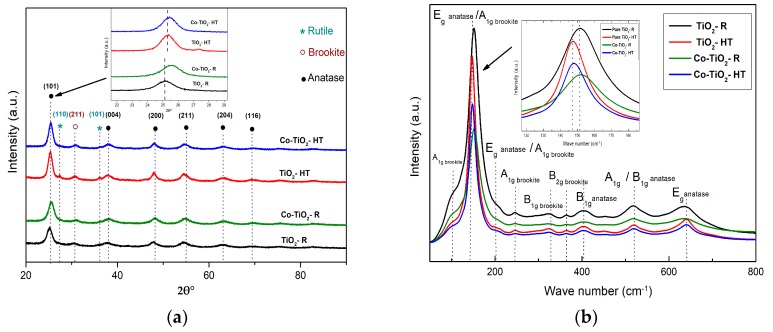
(**a**) X-ray diffraction (XRD) patterns, and (**b**) Raman spectra of TiO_2_-R, Co-TiO_2_-R, TiO_2_-HT, and Co-TiO_2_-HT composites.

TiO_2_ and Co-doped TiO_2_ NPs were synthesized by thermal treatment of solutions of the Ti- and Co-precursors in propanol/water in an open system at the boiling point (reflux) and in a closed autoclave at 200 °C. The NPs obtained by the reflux method were labeled as TiO_2_-R and Co-TiO_2_-R, while the materials obtained at 200 °C were labeled as TiO_2_-HT and Co-TiO_2_-HT. The structural parameters and the phase purity of Co-TiO_2_-R, Co-TiO_2_-HT, TiO_2_-R, and TiO_2_-HT were investigated by means of powder X-ray diffraction (XRD). Powder XRD patterns of all prepared photocatalysts are plotted in Figure 1a.

All materials exhibit diffraction peaks occurring at 2θ = 25.42°, 38.18°, 48.24°, 55.30°, 63.12°, and 69.41°, characteristic of the (101), (004), (200), (211), (204), and (116) planes of anatase TiO_2_ (JCPDS 01-072-4820), respectively. Only trace amounts of brookite TiO_2_ were detected at 2θ = 30.83° in all NPs (JCPDS 01-076-1934). The XRD patterns of pure TiO_2_-HT show weak peaks at 2θ = 27.39° and 2θ = 36.08°, which were attributed to the (110) and (101) planes of the rutile phase (JCPDS 01-089-0552), respectively. There were no other TiO_2_ peaks or any peaks that could be ascribed to Co, CoO, or CoTiO_3_, which is consistent with highly orientated Co-TiO_2_ without any impurity phase. The particle sizes were calculated from the XRD data using the Debye Scherrer equation, and the calculated values are shown in Table 1. The cobalt doping seems to decrease the particle size of the Co-TiO_2_-HT, whereas it does not affect the particle size of the Co-TiO_2_-R.

The Raman spectra of Co-TiO_2_-R, Co-TiO_2_-HT, TiO_2_-R, and TiO_2_-HT composites measured at room temperature in the range between 80 cm^−1^ and 800 cm^−1^ are shown in Figure 1b. All samples reveal Raman bands at around 148, 402, 519, and 639 cm^−1^ associated with E_g_, B_1g_, A_1g_ or B_1g_, and E_g_ vibrations of anatase TiO_2_, respectively. The additional very weak signal at 197 (sh) cm^−1^ was also resolved and assigned to anatase TiO_2_. Characteristic peaks of brookite titania at 245, 324, and 364 cm^−1^ were also observed. In order to clearly see the changes associated with cobalt doping, the full widths at half-maximum (FWHM) of the bands were calculated and given in Table 2. A slight change in the Eg signals of anatase phase in all FWHM Raman signal values towards higher values indicates deviations in the local structure around Ti^4+^ after cobalt modification, thus indicating the incorporation of cobalt ions in the TiO_2_ lattice.

**Figure 2:**
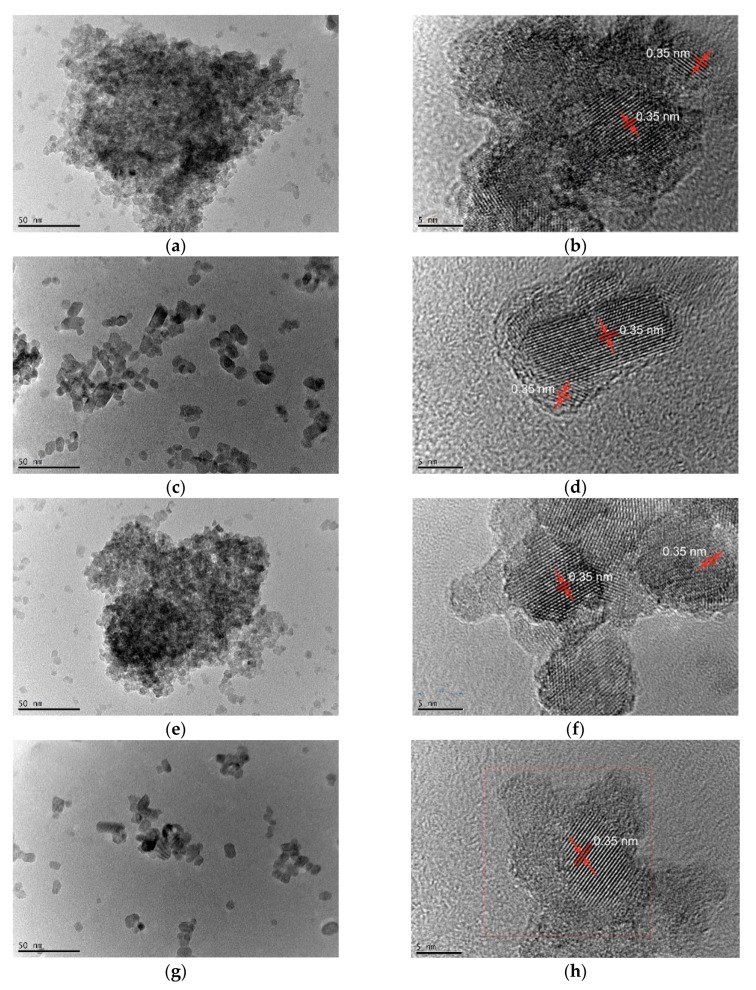
TEM and HRTEM images of (**a**,**b**) TiO_2_-R, (**c**,**d**) TiO_2_-HT, (**e**,**f**) Co-TiO_2_-R, and (**g**,**h**) Co-TiO_2_-HT composites.

Morphological and detailed structural features of Co-TiO_2_ and TiO_2_ NPs were further explored using transmission electron microscopy (TEM) and high-resolution transmission electron microscopy HRTEM. Figure 2a,c,e,g show TEM images of Co-TiO_2_ and bare TiO_2_. The TiO_2_ NPs appear transparent and dense when the particles are in layers. The HRTEM images in Figure 2b,d,f,h show that all materials are well crystallized, as indicated by the spacing of 0.35 nm, which corresponds to the (101) plane of anatase TiO_2_. The crystallite sizes of TiO_2_-R, Co-TiO_2_-R, TiO_2_-HT, and Co-TiO_2_-HT derived from the TEM images are in good agreement with the sizes calculated from the XRD data with the Scherrer equation (Table 1).

**Figure 3:**
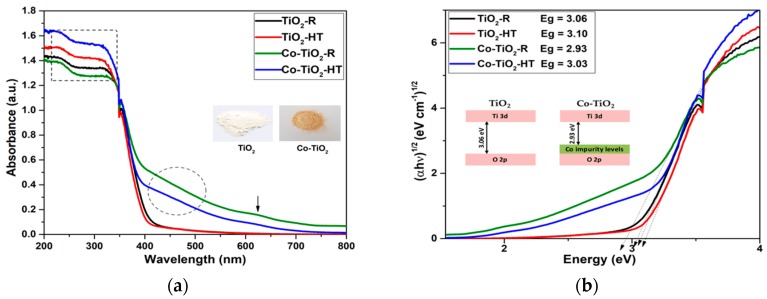
(**a**) UV/vis diffuse reflectance spectra and (**b**) the corresponding indirect band gap energies of TiO_2_-R, Co-TiO_2_-R, TiO_2_-HT, and Co-TiO_2_-HT nanoparticles (NPs).

The light absorption property of the as-prepared photocatalysts was explored through UV/vis diffuse reflectance spectroscopy measurements. The corresponding results are depicted in Figure 3a. The weak peak around 350 nm is the result of the switching in the absorption of the lamp. TiO_2_-HT and TiO_2_-R exhibit no optical response in the visible region. Cobalt doping into TiO_2_ lattice resulted in a significant absorption in the visible region (400–700 nm) of the spectrum. The band gap energies are obtained by converting the UV/vis absorbance spectra into Tauc plots using the equation (αħν)^1/n^ = A(ħν-Eg), where ħ is Planck’s constant, ν is the frequency, α is the absorption coefficient, and A is a proportionality constant. The value of the exponent n denotes the nature of the transition, which is equal to 1/2 for a direct band gap or equal to 2 for an indirect band gap transition. The plots shown in Figure 3b indicate indirect allowed transitions in all four NPs, with band gap energies as given by the intercept of the tangent lines with the abscissa in Figure 3b of 2.93–3.03 eV and 3.06–3.10 eV for the Co-doped and the bare TiO_2_ NPs, respectively (Table 1).

### 2.2. Photocatalytic Activities of Co-TiO_2_-R and Co-TiO_2_-HT on UV/Vis Light-Induced OTC HCl Degradation

**Figure 4:**
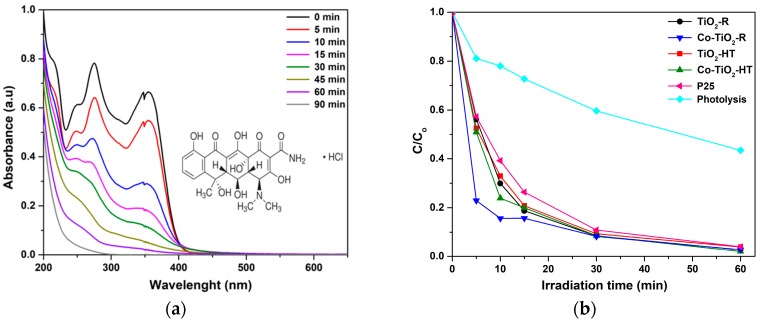
(**a**) Time-dependent UV/vis spectra of oxytetracycline hydrochloride (OTC HCl) solution at pH = 5, and (**b**) kinetics of OTC HCl (30 mg·L^−1^; pH = 5) photodegradation using Co-TiO_2_ and TiO_2_ photocatalysts upon UV/vis illumination.

The photocatalytic ability of the synthesized photocatalysts was evaluated in the aqueous phase at constant pH (pH 5) by the photodegradation of OTC HCl as the target pollutant under the full output of a xenon arc lamp (UV/vis illumination). The intensity of the OTC HCl characteristic peak decreased with increasing the UV/vis irradiation time, as shown in the time-dependent UV/vis absorbance spectra in Figure 4a. The experimental results of the photocatalytic degradation over all samples are depicted in Figure 4b. To further compare the photocatalytic activities of the cobalt-doped and bare TiO_2_ materials, kinetic analysis of OTC HCl photodegradation was carried out by assuming first-order kinetics; ln (C_t_/C_0_) = −kt. The corresponding initial reaction rates were calculated (r_0_ = k × C_0_) and are shown in Table 1. Among all prepared composites, the catalyst prepared by the reflux synthesis showed a higher initial rate of OTC degradation (8.83 mg·L^−1^·min^−1^). As depicted in the table, the observed initial reaction rates for the bare TiO_2_ and Co-doped TiO_2_ materials were found to decrease in the following order: Co-TiO_2_-R > Co-TiO_2_-HT > TiO_2_-HT > TiO_2_-R > P25.

## 3. Discussion

### 3.1. Characterization of Co-TiO_2_-R and Co-TiO_2_-HT Composites

The effect of the preparation method as well as the effect of cobalt ions doping on TiO_2_ for the photodegradation of oxytetracycline hydrochloride pharmaceutical was investigated in this study. The highest photocatalytic activity was achieved for the catalyst modified with 0.5 wt.% cobalt and prepared in reflux (i.e., Co-TiO_2_-R; Figure 4b). It is known that dopant atoms may be introduced in TiO_2_ either substitutionally or interstitially depending on the ionic radius of the dopant. As previously reported by Rodríguez-Talavera, et al. [65], the substitution of high spin Co^2+^ with the ionic radius R(Co^2+^) = 0.885 Å for the octahedral Ti^4+^ with the ionic radius R(Ti^4+^) = 0.745 Å in the TiO_6_ octahedra of the anatase structure induces O^2−^ vacancies and might cause the lattice distortion to rise. The XRD patterns of the Co-TiO_2_ resemble those of the bare TiO_2_ without any peaks associated with metallic Co or cobalt oxides, confirming that cobalt is present as Co (II) ions. The structural characteristic of Co-TiO_2_ and bare TiO_2_, as displayed in Figure 1a, are mainly composed of the anatase phase. The presence of small contamination of the brookite phase is evidenced in all samples at 2θ = 30.83°, with a possible overlapping of the (120) and (111) peaks of brookite at 2θ = 25.34° and 25.69° with the (101) diffraction peak of anatase at 2θ = 25.28°. The appearance of brookite was further confirmed by the analysis of Raman spectra (Figure 1b). This observation of the brookite phase is probably because of the acidic conditions in which the synthesis was performed, as seen in the work of [66], and since, it has been reported by the authors of [67] that the synthesis in ammonia limits the brookite formation. In the TiO_2_ prepared under hydrothermal conditions, two small diffraction peaks characteristic of the rutile phase were evidenced at 2θ = 27.39° and 2θ = 36.08°, indicating lower stability of this sample, which was not seen in the case of bare TiO_2_-R. No rutile phase was detected for Co-TiO_2_-R and Co-TiO_2_-HT, which can be explained by the stabilizing effect of Co ions on the crystalline structure of anatase TiO_2_, preventing the formation of the rutile phase. This observation was also previously reported in Co-doped TiO_2_ [46]. Additionally, the position of the most intense peak and the lattice parameters of anatase phase (101) for the Co-TiO_2_-R and Co-TiO_2_-HT samples are significantly shifted towards a higher angle, as shown in Figure 1a (inset), supporting the substitution of some Co ions into the Ti lattice site. This Co substitution has also been described by Le, et al. [68]. The particle sizes of the synthesized photocatalysts as listed in Table 1 remain almost unchanged for the catalysts prepared in reflux (9.5 ± 0.2 nm and 9.2 ± 0.2 nm), whereas the crystallite size of Co-TiO_2_-HT is slightly smaller than that of the TiO_2_-HT sample (9.9 ± 0.2 nm and 8.4 ± 0.2 nm). It is generally expected that the crystallite size decreases after metal doping, which may be explained by the Co-O bond formation on the surface of the modified titania, which might be responsible for the non-growth of TiO_2_ crystallite.

The Co-induced structural modification of TiO_2_ NPs was further analyzed with micro Raman spectroscopy, which is shown in Figure 1b. In addition to the common anatase vibrations, a weak sub-band at 197 cm^−1^, which may coincide with the brookite band (A1g), and a very weak signal at 447 cm^−1^, characteristics of the rutile phase, were observed in all materials. The reason for not detecting the brookite characteristic peak at 151 cm^−1^ could be because of the overlapping with the anatase intense band (Eg), which is also observed at about 148 cm^−1^. These results are in good agreement with the XRD data and match well with those reported for anatase and brookite phases of titania [69]. As is well known, doping TiO_2_ with Co^2+^ ions induces the formation of oxygen vacancies, because substitution of Ti^4+^ by Co^2+^ demands oxygen vacancy to balance the charges [65]. A very close look into the spectra in Figure 1b (inset) reveals a small shift in the most intense Raman band (Eg) at 148 cm^−1^. This result suggests that Co^2+^ ions were inserted into the anatase structure and substituted the Ti ions in the crystal lattice. Moreover, the intensity of the signals in the Co-doped samples has decreased, indicating the effect of the cobalt atoms on the lattice vibration of titania. Similar behavior has been reported by Huang, et al. for the 5 at.% Co-doped TiO_2_ nanotubes prepared by sol/gel [47]. This shift in the anatase/brookite peak position (Eg) with the decrease of the peak intensity and the change in FWHM values of Co-TiO_2_-R and Co-TiO_2_-HT endorse the incorporation of cobalt ions in the TiO_2_ matrix. No vibration modes for cobalt clusters or cobalt oxides were observed, which additionally supports the presence of dopant cation in the substitutional positions of the titania host lattice in the Co-TiO_2_ NPs. These outcomes are well consistent with the XRD results.

To further elucidate the size and the structure of the Co-doped TiO_2_ NPs, TEM measurements were carried out and are presented in Figure 2. Owing to the low amount of the dopant (0.5 wt.%), defects in the lattice structures do not become observable. As revealed by the HRTEM results in Figure 2b,d,f,h, the interlayer distance of the NPs is about 0.35 nm for TiO_2_-R, Co-TiO_2_-R, TiO_2_-HT, and Co-TiO_2_-HT, which is assigned to the crystal plane (101) of anatase TiO_2_. As indicated in Figure 2c,g using the hydrothermal method, TiO_2_ was transformed and appears as well-defined multilayer spherical and pseudo-cubic in shape, which could be probably because of the small amount of brookite phase present in the titania [70]. The lattice fringes obtained with an interval of 0.35 nm, thus corresponding to the (101) plane of anatase and the (210) plane of brookite [52,70], imply that the NPs are highly crystalline, which is in accordance with the XRD patterns and Raman shifts shown in Figure 1. From these TEM images, the average particle size of the as-synthesized NPs is found to be about 9.8, 9.5, 10.5, and 9.7 nm, and almost independent from the synthetic method within the limit of the experimental error (±0.2 nm) for TiO_2_-R, Co-TiO_2_-R, TiO_2_-HT, and Co-TiO_2_-HT, respectively. These particle sizes are in good agreement with published data [59], and approximately in the same range with the particle sizes calculated from the XRD data using Scherrer’s formula (Table 1).

The specific surface area (SSA) is assumed to play a crucial role in photocatalytic reactions. The SSA of all synthesized photocatalysts was measured via BET adsorption analysis and the values obtained for the Co-doped TiO_2_ and bare TiO_2_ samples are summarized in Table 1. The surface areas of the synthesized TiO_2_-R and Co-TiO_2_-R composites are almost identical considering the experimental error of the device, giving the values of (160 ± 5) and (153 ± 5) m^2·^g^−1^ for TiO_2_-R and Co-TiO_2_-R, respectively. However, doping TiO_2_ with cobalt using the hydrothermal route resulted in a slight increase of the surface area from (109 ± 5) up to (126 ± 5) m^2^·g^−1^, which was also reported in the work of [71]. It is reasonable to assume that Co doping affects the TiO_2_ unit cell parameters, resulting in a distortion of the crystal lattice, which may increase the surface area of the doped material, reflecting a loss of the crystallinity. However, the surface area of Co-TiO_2_-R remains greater than that of Co-TiO_2_-HT, allowing the assumption that Co-TiO_2_-R adsorbs more substrate than Co-TiO_2_-HT.

The optical properties of pure TiO_2_ and Co-TiO_2_ prepared by hydrothermal and reflux methods were investigated. The results depicted in Figure 3a indicate that doping TiO_2_ with cobalt significantly increases the light absorbance of the materials. The diffuse reflectance spectra of pure TiO_2_ consist of a sharp absorption edge around 400 and 407 nm for TiO_2_-HT and TiO_2_-R, which is attributed to the electron transition from the valence band to the conduction band O_2p_-to-Ti_3d_, whereas the Co-doped samples have an extended visible light absorption range with absorption bands up to 410 and 423 nm for Co-TiO_2_-HT and Co-TiO_2_-R, respectively. It is also worth noting that Co-TiO_2_-HT, as seen from the framed region in the UV region of Figure 3a, has the highest absorbance intensity in the UV spectrum, followed immediately by bare TiO_2_-HT. Furthermore, the UV/vis absorbance spectra of the Co-doped materials exhibit a tail in the visible range from 400 nm to 700 nm. The additional broad absorption band in the region between 420 and 520 nm (marked with a circle) may be assigned to the ^4^T_1g_-to-^4^T_1g_ (P) transition, and the weak peak at 620 nm (marked with an arrow) can be attributed to the ^4^T_1g_-to-^4^A_2g_ transition for high spin Co^2+^ (3d^7^) incorporated into the TiO_2_ framework, as previously reported in the literature [27,59,72,73]. From the photocatalysis viewpoint, this sub-band level within the band gap is of great use, as it can be possible to tune the absorption onset to the higher visible wavelength by Co-doping and improve the photocatalytic activity.

The determination of the corresponding band gaps of the pure TiO_2_ and Co-doped TiO_2_ samples was evaluated using the Tauc plot method. The (αħν)^1/n^ versus (ħν) plots of the catalysts are presented in Figure 3b. Plotting (αħν)^1/n^ versus (ħν) is a matter of testing n = 1/2 or n = 2 to compare which gives the better fit, and thus identifies the correct electron transition type occurring in Co-TiO_2_ powders. In TiO_2_-R and TiO_2_-HT materials, the square power (αħν)^1/2^ used as titanium dioxide is well known to have an indirect allowed transition. Thus, the optical absorption band gaps (Eg) for TiO_2_-R and TiO_2_-HT were estimated to be 3.06 and 3.10 eV, respectively. In order to find out the effect of the cobalt ion doping on the TiO_2_ band gap, the Tauc plots of the Co-TiO_2_ samples were also analyzed and depicted in Figure 3b. As could be seen, Co-TiO_2_-R exhibited band gap energy, which apparently decreased up to 2.93 eV, possibly because of the generation of Co-3d defect states near the valence band maximum of TiO_2_-R, as shown in the inset of Figure 3b, whereas the cobalt doping using hydrothermal synthesis does not affect significantly the band gap of TiO_2_-HT with a value of 3.03 eV for Co-TiO_2_-HT. This narrowing of the energy band gap of Co-TiO_2_ has been also observed by the authors of [59], Choudhury [48], and Khurana, et al. [49], and was explained by the introduction of new impurity states near the valence band edge of TiO_2_. It can be concluded from these results that Co^2+^-doping employing reflux synthesis results in a remarkable decrease in the band gap of TiO_2_ and a red shift of the absorption onset within the visible spectrum, leading to much greater electrons and holes generation, which could migrate to the surface to drive redox reactions with the adsorbed pharmaceutical.

### 3.2. UV/Vis Light-Induced Oxytetracycline Hydrochloride Degradation over Co-TiO_2_ Composites

The solar light-induced photocatalytic ability of the synthesized Co-doped TiO_2_ system was evaluated through the degradation of OTC HCl in aqueous suspension at constant pH. A comparison of the activity was made with undoped TiO_2_ and the commercial Degussa P25. The UV/vis light-induced degradation profile of OTC HCl using pure TiO_2_ and Co-doped TiO_2_ is given in Figure 4b. As previously reported [74,75], OTC HCl has four species at different pH ranges, and each species has a unique electric charge state, which may have an influence on the photolytic and photocatalytic degradation under both UV and visible light. To avoid any possible changes in the form of OTC HCl, it was chosen to maintain the pH at 5, which corresponds to the neutral zwitterions form H_2_OTC^±^. Under UV/vis irradiation, the light-induced degradation of OTC HCl in terms of initial reaction rates was found to increase drastically from 3.45 to 8.83 mg·L^−1^·min^−1^ for the Co-TiO_2_-R composite. A negligible increase in the initial rates from 3.87 to 4.05 mg·L^−1^·min^−1^ was also observed for the Co-TiO_2_-HT. Furthermore, the observed initial reaction rate for all composites was higher than that of the commercially available P25. These experimental results agree well with the BET surface area results and the UV-vis absorption data, suggesting that the high surface area along with the reduced band gap of the Co-TiO_2_-R have a major influence on the kinetics of the photocatalytic performance of this material (Table 1). It is also worth noting that all samples led finally to complete mineralization of OTC HCl, even bare TiO_2_. This may possibly be because of the mixed brookite with anatase phases leading to an occurrence of junctions among different polymorphic TiO_2_ phases that enhance the separation of the photogenerated electron–hole pairs under UV light. It is also expected that, with UV/vis, excitation of TiO_2_ using a xenon arc lamp suggests that photocatalysis acts by direct near-UV excitation of TiO_2_. Additionally, and owing to the photosensitization reaction, bare TiO_2_ could be rather activated under visible irradiation.

Comparing the photocatalytic results obtained with the composites prepared by the two synthesis methods, the UV/vis light-induced degradation of OTC HCl mediated by Co-TiO_2_ and TiO_2_ using the hydrothermal synthesis seems to have an increasing function with the BET surface area. Compared with TiO_2_-HT, Co-TiO_2_-HT has a significantly larger specific surface area and smaller particle size, which indicates that the Co species evidently decelerates the crystal-growth rate of the anatase phase. This result suggest that cobalt doping can significantly increase the specific surface area for the Co-TiO_2_-HT sample and prevent the phase transformation of anatase to rutile phase, which was evidenced by the absence of the two main peaks of rutile at 2θ = 27.39° (110) and 2θ = 36.08° (101) observed in the XRD patterns of TiO_2_-HT, resulting in the stability of the material with a slight enhancement of the photocatalytic activity of the light-induced degradation of OTC HCl using Co-TiO_2_-HT. Controversially, the Co-doping did not significantly affect the specific surface area of Co-TiO_2_ synthesized under reflux. However, the surface area of Co-TiO_2_-R stayed larger than that of Co-TiO_2_-HT. On the other hand, Co-TiO_2_-R showed the highest UV/vis light absorption, resulting in efficient UV/vis light-induced degradation of the target pollutant. This high enhancement seen in the case of the co-doped sample prepared by the reflux method could be attributed in part to its high surface area, which allows to adsorb more substrate (OTC HCl) on the surface of the catalyst, and in another part to the high ability of this catalyst to absorb UV/visible light, which facilitates the electron–hole pair generation participating in the photocatalytic reactions in the system.

The blank experiment indicates that the direct photolysis of OTC HCl cannot be ignored, because around 38% was decayed without a photocatalyst within 30 min of UV/vis irradiation. This result suggests the responsible mechanism for the photolytic degradation of OTC HCl at pH 5, which may involve the excitation of OTC HCl to singlet (S_1_) or triplet (T_1_) states (OTC*) under UV light.

### 3.3. Proposed Mechanisms of UV/Vis Light-Induced Oxytetracycline Hydrochloride Degradation Using Co-TiO_2_ Catalysts

In general, the photocatalytic oxidation of organic compounds mainly involves the photo-absorption of the photocatalyst, the generation of photogenerated electron–hole, the transfer of charge carriers, and the consumption of the charge carriers by the targets [76]. Co-doped TiO_2_ materials showed the ability to absorb visible light, affecting the transition of the electrons from the VB (O 2p) to CB (Ti 4d) in the photocatalysts. Figure 3a showed that the light absorption by the Co-doped TiO_2_ samples occurred mainly when λ < 400 nm. As shown in Figure 4a, OTC HCl has no light absorption characteristics in the visible region (wavelengths longer than 400 nm) and was degraded within a certain wavelength range of the UV light. However, it was found that OTC HCl was decayed by Co-TiO_2_ materials upon UV/visible-light irradiation. Thus, the materials absorbing light irradiation in the present study are supposed to be Co-TiO_2_ catalyst and OTC HCl. Therefore, both photocatalytic and photosensitized process would work simultaneously under these experimental conditions.

As bare TiO_2_ absorbs only UV light, the degradation of OTC HCl can be induced indirectly by the absorption of the TiO_2_ conduction band electron acting as an electron scavenger on the TiO_2_ surface. In addition to the photocatalytic oxidation mechanism, which is thermodynamically possible, the photosensitizing oxidation mechanism of OTC HCl can also occur, suggesting that the electron from the excited OTC HCl molecule is injected into the conduction band of the TiO_2_, and the radical formed at the surface rapidly undergoes degradation to yield products (Equations (1) and (2)) [74,77]. The photocatalytic degradation of OTC HCl over bare TiO_2_ might also occur by the combined action of holes and OH^•^ yielding products.

OTC HCl + hυ → OTC*(1)

OTC* HCl + TiO_2_ → OTC HCl + TiO_2_ (e^−^) (2)

**Figure 5:**
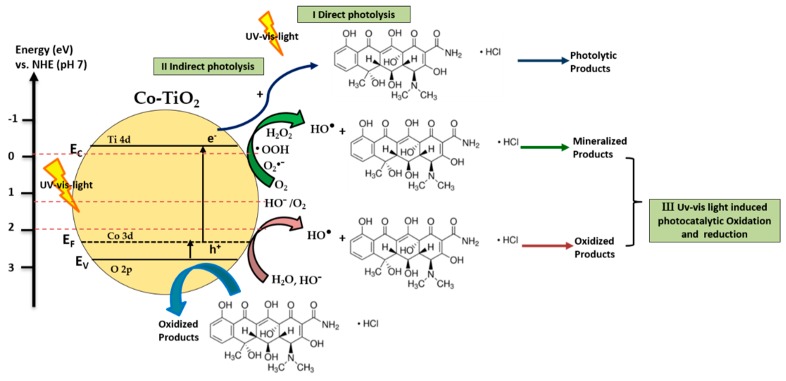
Illustration of the mechanism of UV/visible light-induced OTC HCl degradation using Co-TiO_2_ NPs.

Considering the present results, a mechanism of the light-induced charge transfer behaviors during the degradation of OTC HCl and using the Co-doped TiO_2_ is illustrated in Figure 5. Accordingly, the cobalt would introduce a new energy level (3d orbit) just above the valence band of TiO_2_ and decreases the band gap, as shown in Figure 3b. Hence, Co-TiO_2_-R and Co-TiO_2_-HT can be activated under visible light. Therefore, more electrons from the visible region are used to produce photogenerated electrons and holes. The photogenerated electrons accumulated in the Co-TiO_2_ conduction band could easily transfer to the adsorbed oxygen O_2_, forming a superoxide radical anion O_2_^•−^ (−0.13 V vs. NHE), which combines with H^+^ to form hydrogen peroxide H_2_O_2_ (0.89 V vs. NHE). Consequently, O_2_^•−^ reacts with H_2_O_2_, generating OH• (0.38 V vs. NHE), which further converts OTC HCl to mineralized products. On the other hand, the photogenerated holes (h^+^) accumulate in the valence band of Co-TiO_2_ and either oxidize directly the pollutant, or they are consumed by participating also in the oxidation of water yielding OH• (1.89 V vs. NHE), which further oxidizes OTC HCl. The Co species could trap part of the photogenerated holes. Thus, the recombination rate of photogenerated electrons and holes might be decreased. Consequently, the photocatalytic degradation efficiency of OTC HCl over Co-TiO_2_ catalysts is improved (Figure 5).

## 4. Materials and Methods

### 4.1. Materials’ Composites

Titanium (IV) isopropoxide (Ti (OPri)_4_ (97%)), cobalt (II) acetate tetrahydrate (Co (Ac)_2_∙4H_2_O (99.99%)), oxytetracycline chloride (OTC HCl, 95%), and hydrochloride acid (HCl, 37%) were purchased from Sigma Aldrich Chemie GmbH, München, Germany. 2-Propanol anhydrous (99.5%, Carl Roth GmbH, Karlsruhe, Germany) and methanol (99.9%, Carl Roth GmbH, Karlsruhe, Germany) were of analytical grade and used without further purification. Aeroxide TiO_2_ P25 with a mixture of anatase (80%) and rutile (20%) crystal phase and a specific surface area of 50.1 m^2·^g^−1^ was kindly provided by Evonik Industries AG, Essen, Germany. Deionized water from a Sartorius Arium 611 device (Sartorius AG, Göttingen, Germany) with a resistivity of 18.2 MΩ·cm was used for the preparation of all aqueous solutions.

### 4.2. Photocatalysts Synthesis

#### 4.2.1. High Temperature Synthesis of Cobalt-Doped TiO_2_

Two solutions were prepared: solution A containing a prescribed amount of titanium isopropoxide (Ti (OPri)_4_) as the TiO_2_ precursor dissolved in anhydrous propanol with vigorous stirring. Solution B was prepared by adding 0.5 g Co (Ac)_2_∙4H_2_O to 50 mL of 2-propanol, 10 mL of distilled water, and 0.5 mL of 1M HCl in a 500 mL flask and stirring for 20 min. Solution A was then added dropwise to solution B with continuous stirring. The formed gel was aged for 24 h to ensure complete hydrolysis. The obtained mixture was transformed into a stainless-steel autoclave and hydrothermally heated to 200 °C for 10 h. After the autoclave was cooled to room temperature, the yellow precipitate at the bottom of the autoclave was separated, washed with ethanol and deionized water several times, and dried at 70 °C overnight. The obtained residue was calcined at 500 °C for 5 h and denoted as Co-TiO_2_-HT. For comparison purposes, the pure TiO_2_ was synthesized using the same procedure without adding the Co precursor. Bare TiO_2_ catalyst was denoted as TiO_2_-HT.

#### 4.2.2. Reflux Synthesis of Cobalt-doped TiO_2_

In order to synthesize the same photocatalysts by the reflux method, the mixture of solutions A and B were refluxed for 6 h. The obtained residue was calcined at 500 °C for 5 h and denoted as Co-TiO_2_-R. The pure TiO_2_ was prepared using the same procedure without adding the cobalt precursor. The pure TiO_2_ catalyst was denoted as TiO_2_-R.

### 4.3. Photocatalysts Characterization

The crystalline structure of the Co-TiO_2_-R, Co-TiO_2_-HT, TiO_2_-R, and TiO_2_-HT catalysts was measured by powder X-ray diffraction (XRD) (D8 Advance system, Bruker, Billerica, MA, USA), using a Cu Kα radiation source with a wavelength of λ = 1.54178 Å over a 2θ range from 20° to 100°, with a 0.011° step width. The average crystal sizes of the synthesized Co-TiO_2_ and safe TiO_2_ samples were calculated by applying the scattering characteristic of the anatase structure to the Scherrer equation. Raman measurements were made employing a confocal micro-Raman spectrometer (Senterra Bruker Optik GmbH, Ettlingen, Germany). All depolarized spectra were obtained at ambient conditions in backscattering geometry using an Olympus BX 51 microscope (Olympus Corp., Tokyo, Japan) that allows the incident 532 nm laser beam to be focused on the sample as a spot of about 2 µm in diameter. An integration time of 1 s, 50 co-additions, and a power of 2 mW were used. The instrumental precision was within ±3 cm^−1^. Diffuse reflectance (DR) UV/vis spectroscopy was employed using a spectrophotometer (Varian Spectrophotometer Cary-100 Bio, Agilent Technologies, Santa Clara, CA, USA) at room temperature. Barium sulfate (BaSO_4_) was used as a standard for 100% reflectance measurement. Reflectance was converted by the instrument software to F[R] values according to the Kubelka–Munk theory. The specific surface area (SSA) of the investigated materials was determined according to the multi-layer adsorption model by the Brunauer–Emmet–Teller (BET) method using a FlowSorb II 2300 apparatus from Micromeritics Instrument Company (Norcross, GA, USA). Prior to all measurements, the samples were evacuated at 180 °C for 1 h. Transmission electron micrographs (TEMs) of the catalysts were taken by means of a TEM Tecnai G2 F20 TMP device (FEI Company, Hillsboro, OR, USA) operated at an acceleration potential of 200 kV with an FEG field effect, objective lenses TWIN, and point resolution of 0.27 nm.

### 4.4. UV/Vis Light-Induced Oxytetracycline Hydrochloride (OTC HCl) Degradation

**Figure 6:**
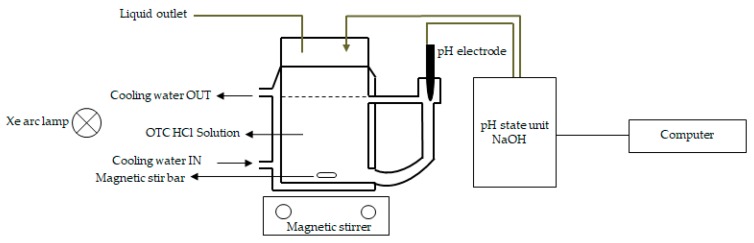
Scheme of the experimental set-up used for the photocatalytic degradation experiments.

Photocatalytic efficiencies of the commercial Degussa P25, pure TiO_2_, and Co-TiO_2_ photocatalysts were measured for oxytetracycline hydrochloride (OTC HCl) photodegradation as a model compound. The photocatalytic degradation experiments were carried out using a 300 W Xenon arc lamp (Müller Electronik-Optik, Moosinning, Germany) as the UV/vis light source. The experiments were conducted on a Pyrex glass reactor with a capacity of 230 mL and equipped with a cooling jacket. The temperature was maintained constant at 25 °C using a thermostatic bath (Julabo GmbH, Seelbach, Germany). An aqueous solution of OTC HCl (230 mL, 30 mg·L^−1^) and 0.5 g·L^−1^ of the catalyst were added to the Pyrex reactor and stirred for 30 min before starting the degradation experiment in order to reach maximum output. The pH of the solution was adjusted to pH = 5 by adding solutions of HNO_3_ and NaOH using a pH-stat technique. This technique consists of an automatic dosing unit (Basic Titrino 794 from Metrohm AG, Herisau, Switzerland) with a high-performance titrimetric pipette able to add drops of 0.5 μL, a highly sensitive semi-micro pH electrode combined with an Ag/AgCl reference electrode (Thermo-Orion Ross 8115, Chelmsford, UK) with pH accuracy up to the third decimal, and a computer to control and register the results. The system was then irradiated by a 300 W Xenon arc lamp for 90 min. The entire experimental set-up is shown in Figure 6. Aliquots (1.5 mL) were withdrawn periodically, centrifuged to remove the catalyst, and analyzed immediately. Two independent analytical methods were used, that is, UV/vis analysis and high-performance liquid chromatography (HPLC) (Ecom System Inc., Sarasota, FL, USA) equipped with UV/vis detectors operated at 355 nm and a Knauer Vertex plus column packed with Eurospher II 100-5 C18 A material (L × I.D. = 150 cm × 4 mm) with precolumn. The oven temperature was 30 ˚C and the mobile phase was a mixture of methanol/acetonitrile/(0.01 mol/L) oxalic acid solution (20/20/60, *v/v*%).

## 5. Conclusions

To summarize, Co-TiO_2_ composites synthesized by means of reflux and hydrothermal methods were found to enhance the light-induced degradation rate of OTC HCl under UV/vis irradiation. Significant differences in the structural analysis were observed between the materials prepared by the two preparation methods. Co-doping through the hydrothermal synthesis resulted in preventing the rutile phase formation with an increase in the surface area, while doping using the reflux method does not affect the specific surface area. The Co-doped TiO_2_ NPs prepared by reflux showed enhanced UV/vis light-induced OTC HCl degradation with an initial rate of 8.83 mg·L^−1·^min^−1^, which was higher than the degradation rate of the all prepared catalysts and commercial P25. The high photocatalytic activity of the Co-TiO_2_-R was attributed to its high surface area, which serves as a good absorber for the substrate molecule, and to the defect levels created below the valence band of TiO_2_-R, which lead to better charge separation and improve the kinetic properties of this material. Co-TiO_2_-assisted photodegradation of OTC HCl was found to occur via two competitive processes: a photocatalytic process and a photosensitized process. In the photocatalytic process, direct hole transfers, O_2_•ˉ, and OH• could take part in the Co-TiO_2_ photocatalysis.

## Figures and Tables

**Table 1:** The crystal size, specific surface area (SSA) values, apparent band gap, and initial rates (r**_0_**) of oxytetracycline hydrochloride (OTC HCl) degradation in the presence of TiO_2_-R, Co-TiO_2_-R, TiO_2_-HT, Co-TiO_2_-HT, and commercial P25. TEM, transmission electron microscopy.

Catalysts	XRD Size (nm)	TEM Size (nm)	SSA (m^2^·g^−1^)	Band Gap (eV)	r_0_ [OTC HCl] UV/vis (mg·L^−1·^ min^−1^)
TiO_2_-R	9.5	9.8 ± 0.2	160 ± 5	3.06	3.45
Co-TiO_2_-R	9.2	9.5 ± 0.2	153 ± 5	2.93	8.83
TiO_2_-HT	9.9	10.5 ± 0.2	109 ± 5	3.10	3.87
Co-TiO_2_-HT	8.4	9.7 ± 0.2	126 ± 5	3.03	4.05
P25	21	20.0 ± 0.2	50 ± 5	3.06	3.34
Photolysis	-	-	-	-	1.26

**Table 2:** The full widths at half-maximum (FWHM) of TiO_2_-R, Co-TiO_2_-R, TiO_2_-HT, and Co-TiO_2_-HT composites.

Catalysts	E_g_ Anatase	A_1g_ Brookite	B_1g_ Brookite	B_2g_ Brookite	B_1g_ Anatase	A_1g_/B_1g_ Anatase	E_g_ Anatase
TiO_2_-R	32.8	41	75.5	26	93.5	96.5	129.2
Co-TiO_2_-R	34.8	46.5	71	23.5	96	94.5	172
TiO_2_-HT	19.5	33.5	83	27	97	97	84.8
Co-TiO_2_-HT	20.5	31.5	82	28	96.5	92.5	91.3
